# Global miRNA Expression Profiling Identifies miR-1290 as Novel Potential oncomiR in Laryngeal Carcinoma

**DOI:** 10.1371/journal.pone.0144924

**Published:** 2015-12-22

**Authors:** Joanna Janiszewska, Marcin Szaumkessel, Magdalena Kostrzewska-Poczekaj, Kinga Bednarek, Julia Paczkowska, Joanna Jackowska, Reidar Grenman, Krzysztof Szyfter, Malgorzata Wierzbicka, Maciej Giefing, Malgorzata Jarmuz-Szymczak

**Affiliations:** 1 Institute of Human Genetics, Polish Academy of Sciences, Department of Cancer Genetics, Poznan, Poland; 2 Department of Otolaryngology and Laryngological Oncology, University of Medical Sciences, Poznan, Poland; 3 Department of Otorhinolaryngology, Head and Neck Surgery and Department of Medical Biochemistry, Turku University Hospital and University of Turku, Turku, Finland; 4 Department of Audiology and Phoniatry, University of Medical Sciences, Poznan, Poland; 5 Department of Hematology, K. Marcinkowski University of Medical Sciences, Poznan, Poland; University of Cincinnati College of Medicine, UNITED STATES

## Abstract

**Background:**

Laryngeal squamous cell carcinoma (LSCC) is the most common group among head and neck cancers. LSCC is characterized by a high incidence in Europe. With the aim of better understanding its genetic background we performed global miRNA expression profiling of LSCC cell lines and primary specimens. By this approach we identified a cohort of 33 upregulated and 9 downregulated miRNA genes in LSCC as compared to epithelial no tumor controls.

**Results:**

Within this group we identified overexpression of the novel miR-1290 gene not reported in the context of LSCC before. Using a combined bioinformatical approach in connection with functional analysis we delineated two putative target genes of miR-1290 namely *ITPR2* and *MAF* which are significantly downregulated in LSCC. They are interesting candidates for tumor suppressor genes as they are implicated in apoptosis and other processes deregulated in cancer.

**Conclusion:**

Taken together, we propose miR-1290 as the new oncomiR involved in LSCC pathogenesis. Additionally, we suggest that the oncogenic potential of miR-1290 might be expressed by the involvement in downregulation of its target genes *MAF* and *ITPR2*.

## Introduction

Head and neck squamous cell carcinoma (HNSCC) includes a heterogeneous group of tumors, which presents a serious public health concern since they are frequently resistant to treatment, which results in low survival rates. Within HNSCC, laryngeal squamous cell carcinoma (LSCC) is the most common group and accounts for nearly 50% of cases [1, 2]. According to the International Agency for Research on Cancer and European Cancer Observatory (ECO) the age standardized larynx cancer incidence in Europe is 4.4 per 100 000 Europeans [3]. In view of high frequency of laryngeal cancer there is a necessity to better understand its genetic background.

In the last decade, combined DNA copy number and mRNA expression profiling brought insight into the molecular mechanism of LSCC. Several novel oncogenes and tumor suppressors have been delineated contributing to our understanding of HNSCC/LSCC pathogenesis [4–6]. Recently, a new class of non-protein coding genes, namely miRNAs, known to be involved in tumor related processes such as apoptosis or cell cycle regulation has been identified [7]. Previous observations have led to an assumption, that deregulated miRNAs may directly influence downstream protein-coding genes and in consequence function as a novel class of tumor suppressors or oncogenes, which just as their classical counterparts, are frequently located in regions, that are commonly amplified or deleted in human cancers [8, 9]. Moreover miRNAs have a potential to be a novel class of biomarkers detected in body fluids [10].

Several deregulated miRNAs related to HNSCC/LSCC pathogenesis have been identified so far. MiR-21 for example, targeting the *PTEN* tumor suppressor is recurrently overexpressed in HNSCC and correlates with SCC progression [11], whereas miR-155 was shown to downregulate the *CDC73* tumor suppressor gene and inhibit the cytokine signaling 1 (SOCS1)-STAT3 pathway in OSCC (oral squamous cell carcinoma) cell lines [12, 13]. In contrast, downregulation of let-7 in laryngeal cancer correlates with overexpression of *RAS* and *c-MYC* oncogenes and negatively influences the sensitivity to chemotherapy [14]. Besides, the deregulation of several miRNAs including miR-93, miR-375, miR-125b, miR-145, miR-205 and miR-707 was reported recently in HNSCC/LSCC [15–18]. Another important aspect of miRNA deregulation in HNSCC is their association with HPV infection [19].

The aim of presented study was the identification of new deregulated miRNAs and their target genes in LSCC. For this purpose we performed global miRNA expression profiling of laryngeal cancer cell lines and primary specimens and we identified a cohort of deregulated miRNAs that were previously described in the literature. In addition we identified the miR-1290 gene not reported before, to be recurrently overexpressed in LSCC as compared to epithelial no-tumor controls. Moreover, by combining mRNA expression microarray data for the group of LSCC cell lines and no-tumor controls with public available miRNA target databases we indicated potential miR-1290 target genes downregulated in LSCC namely *MAF* and *ITPR2* [20]. Finally, we functionally validated that miR-1290 regulates the expression of *MAF* on mRNA as well as on protein level.

## Materials and Methods

### Cell lines

Twenty cell lines derived from laryngeal squamous cell carcinoma (UT-SCC-4, UT-SCC-6A, UT-SCC-9, UT-SCC-11, UT-SCC-17, UT-SCC-19A, UT-SCC-19B, UT-SCC-22, UT-SCC-23, UT-SCC-29, UT-SCC-34, UT-SCC-38, UT-SCC-50, UT-SCC-57, UT-SCC-75, UT-SCC-106A, UT-SCC-106B, UT-SCC-107, UT-SCC-108 and UT-SCC-116) established at the Turku University, Central Hospital, Finland were used. Cell lines characteristics and culture conditions are described elsewhere [21–23]. These stable larynx cancer-derived cell lines were a kind gift from prof. Reidar Grenman from the University in Turku in Finland.

Additionally HepaRG and HEK293T cell lines were used. Cell cultures were grown in 25 cm^2^ flasks at 37°C under 5% CO_2_ atmosphere. For LSCC cell lines and HEK293T Dulbecco’s modified Eagle medium supplemented with 10% fetal bovine serum was used, while for HepaRG Williams Medium E with 10% fetal bovine serum.

### Primary laryngeal tumor specimens

Fresh frozen tumor samples were obtained from 50 patients (Table 1), all diagnosed with LSCC and undergoing surgical tumor resection followed by radiotherapy in the Department of Otolaryngology, K. Marcinkowski University of Medical Sciences in Poznan, Poland. The Ethics Institutional Review Board at the K. Marcinkowski University of Medical Sciences in Poznan, Poland approved the study (No. 163/10) and an informed consent was obtained in writing from all patients. The tumor tissues were assessed histopathologically to confirm the presence of at least 60% of tumor cells. Within this group 5 samples were used for microarray analyses, 50 for LNA (locked nucleic acid) real-time qPCR and 22 for quantitative real-time PCR.

**Table 1:** Clinicopathological data of the laryngeal tumor specimens.

Parameters	Number	Percent [%]
**Location**		
Larynx	50	100
**Age**		
>60	22	44
<60	28	56
**Gender**		
M	44	88
F	6	12
**Smoking history**		
Yes	34	68
No	1	2
Unknown	15	30
**Grade of tumor**		
Gx	1	2
G1	7	14
G2	34	68
G3	7	14
G4	0	0
Unknown	1	2
**T classification**		
T1	0	0
T2	3	6
T3	18	36
T4	29	58
**Lymph node status**		
N0	18	36
N≠0	32	64
**Metastasis**		
M0	49	98
M1	1	2

### Control group

Control samples for microarray analysis and real-time qPCR included commercially available total RNA derived from healthy human larynx (Stratagene, Basel, Switzerland), human tracheal epithelial cells (HTEC) (PromoCell, Heidelberg, Germany) and human normal bronchial/tracheal mix of epithelial cells (NHBE) (Lonza, Basel, Switzerland). Additionally, two specimens derived from surgical margins assessed histopathologically obtained from K. Marcinkowski Medical University were used as no tumor controls in both LNA real-time qPCR and quantitative real-time qPCR.

### miRNA profiling

#### RNA isolation

Total RNA has been isolated with use of Trizol reagent based on the method developed by Chomczynski described elsewhere [24]. This method allows to obtain all fractions of RNA, including miRNA. RNA quality and quantity was measured by NanoDrop ND100 and A230/260 > 1.8 was considered as appropriate. RNA was also analyzed using the Agilent Bioanalyzer 2100 and RIN (RNA integrity number) > 8 was considered as suitable for further analysis.

#### Microarray analyses

The miRNA expression profiling was performed on Agilent Human miRNA Microarray Expression 60K platform by the Atlas Biolabs GmbH (Berlin, Germany). The array covered 1244 human and 174 viral mature miRNAs (miRbase 16.0 and updates). Together, total RNA samples from 16 LSCC cell lines, 5 primary tumor specimens and 3 no tumor controls were analyzed.

### Validation of microarray results

#### cDNA synthesis

cDNA for miRNA expression analyses was synthesized from total RNA using universal cDNA synthesis kit according to the supplier’s protocol (Exiqon company, Denmark). The kit uses universal method of adding poly-A tails (polyadenylation) thereby allows using 3' degenerate anchors and 5' universal poly-T tag during cDNA synthesis and downstream miRNA- specific and LNA- enhanced primers in amplification.

#### LNA Real-time PCR (miRNA quantification)

Real-time PCR has been conducted on the BioRad iQ5 instrument with SybrGreen Mastermix (Exiqon, Vedbaek, Denmark) and LNA modified primers (Exiqon, Vedbaek, Denmark) for detecting miR-1246 (accession number *MIMAT0005898)*, miR-1290 (MIMAT0005880) and miR-4317 *(MIMAT0016872)*. As reference genes, SNORD44 (Cat. No. 203902) and 5SRNA (Cat. No. 203906) were used as pre-designed primers, while primers for studied miRNA were designed using Exiqon online software (all Exiqon primer sequences are kept confidential by the company).

Real time PCR reactions were performed according to manufacturer's (Exiqon, Vedbaek, Denmark) recommendation for individual assays. Each reaction was performed in triplicate. The BioRad iQ5 Optical System software has generated automatically background values for threshold cycle determination (Ct). Ct values were utilized to calculate the relative expression (in relation to control samples and references genes) of miRNA using the BioRad Genex application.

#### Statistical analysis

Statistical analysis of microarray data was performed by Atlas Biolabs GmbH (Berlin, Germany). The data were transformed into log_2_ ratio and samples compared to controls. The following criteria have been implemented in order to delineate significantly up or downregulated miRNAs: (i) difference in expression of a given miRNA between samples versus controls calculated with t-test with Bonferroni correction must have reached significance level (p<0.05) and (ii) expression fold change of a given miRNA must have differed at least two times between samples and controls.

The data from real-time PCR were analyzed by Mann-Whitney U Test and p<0.05 was considered as significant.

### Identification of miR-1290 target genes

#### Identification of miR-1290 target gene candidates

The miRDB database (http://mirdb.org/miRDB/ [25]) was used to predict target genes for miR-1290. Putative target genes with the probability of interaction (according to miRDB) given as a target score >90 were selected. Additionally, we used the miRWALK software [26], which allows simultaneous searching of 4 databases (miRanda, miRWalk, PICTAR5 and Targetscan) and accepted only the genes listed in at least 2 databases.

MiR-1290 target genes selected with this approach were then analyzed using microarray expression profiles (Affymetrix U133 plus 2.0) for LSCC cell lines published previously [20] to detect genes downregulated in LSCC as compared to epithelial no tumor controls. Target candidate genes which showed reduced expression, according to the formula below, were considered to be potentially regulated by miR-1290: $\frac{level\;of\;expression\;in\;tumor\;samples}{level\;of\;expression\;in\;controls}\leq 0.6(6)$. The data from Affymetrix microarray expression were analyzed by Mann-Whitney U Test and p<0.05 was considered as significant.

#### Functional validation of miR-1290 target genes

Transient transfections of miR-1290 inhibitor (mirVana^®^ miRNA inhibitor, Life Technologies, Carlsbad, CA, USA) were conducted on two cell lines UT-SCC-34, UT-SCC-107 using Lipofectamine^®^ RNAiMAX Transfection Reagent (Life Technologies, Carlsbad, CA, USA) according to manufacturer’s instruction. MirVana^™^ miRNA Inhibitor, Negative Control (Life Technologies, Carlsbad, CA, USA) was used as a negative control of transfection. The cells were transfected by 75 nM of inhibitor/negative control in triplicate on 25 cm^2^ culture flasks and incubated for 48 (UT-SCC-34) and 72 (UT-SCC-107) hours. Additionally, to exclude the influence of transfection reagents on gene expression, we used the untreated cell line UT-SCC-34, UT-SCC-107 as a wild type.

Total RNA was isolated only from UT-SCC-34 cell line (wild type and transfected by inhibitor/negative control) as described above and used for cDNA synthesis. 8 μg of total RNA were reverse-transcribed in a total volume of 40 μl using Maxima First Strand cDNA Synthesis Kit for RT-qPCR (Thermo Scientific Waltham, MA, USA) with dsDNase, according to manufacturer’s instruction.

Total protein was extracted from wild type and inhibitor/negative control transfected UT-SCC-34 as well as UT-SCC-107 cell lines by NP-40 buffer with protease inhibitor cocktail and the protein concentration was determined by Bradford assay (Bio-Rad, CA, USA). In addition total protein was prepared from HepaRG and HEK293T as described above.

#### Quantitative real-time PCR

To verify downregulation of selected genes in LSCC primary samples and influence of miR-1290 on expression level of its target genes, primers were designed and quantitative real-time PCR was performed as previously described (S1 Table) [22]. β-actin was used as the reference gene. Firstly the downregulation of three genes selected on the basis of Affymetrix expression microarray profiles were verified on cDNA from 22 LSCC primary samples and five no tumor controls. Then the expression changes of the selected genes after miR-1290 inhibition were investigated in the UT-SCC-34 cell line. Each reaction was performed in triplicate on the BioRad iQ5 Optical System as described above. Automatically generated background values for threshold cycle determination (Ct) have been used to calculate the relative expression (in relation to control samples and references genes) of selected genes using the BioRad Genex application v1.10. Real-time qPCR data were calculated by Mann-Whitney U Test and p<0.05 was considered as significant.

#### Western blot

The whole cell lysates from wild type and inhibitor/negative control transfected UT-SCC-34 and UT-SCC-107 cell lines were used. Additionally, the cell lysates from HepaRG and HEK293T were used as positive controls for ITPR2 and MAF respectively. Western blot was conducted according to the standard procedure using Mini Protean system (Bio-Rad). The following antibodies were used: rabbit polyclonal anti- MAF antibody (ab77071, Abcam, UK, 1:500), goat polyclonal anti-ITPR2 (sc-7278, Santa-Cruz, TX, USA, 1:500), goat anti-Rabbit, (ab97051, Abcam, UK, 1:25 000) and rabbit anti-Goat (A5420, Sigma, USA, 1:7000). Anti-GAPDH (ab9485, Abcam, UK, 1:2500) or anti-Vinculin (ab129002, Abcam, UK, 1:30 000) antibody was used as a loading control. For protein detection the SuperSignal West Pico Chemiluminescent Substrate (Thermo Scietific, Rockford, IL USA) was used. The images were scanned and analyzed using the ChemiDoc XRS+ System (BioRad).

## Results

### miRNA profiling of laryngeal cancer cell lines and primary samples identifies deregulated miRNAs

By comparing miRNA profiles of sixteen LSCC cell lines with three epithelial no tumor controls we have identified thirty one overexpressed and eight downregulated miRNA genes. Whereas by comparing the five primary larynx cancer specimens with the same three control samples, seven miRNA genes were identified as overexpressed and three were downregulated (p<0.05). Together thirty three overexpressed and nine downregulated miRNAs (each non-unique occurrence was counted only once) were identified (S2 Table).

We next used these cohorts to select miRNAs that were altered both in the cell lines and the primary samples (Table 2). These included five overexpressed (miR-1246, miR-21-5p, miR-21-3p, miR-1290, miR-4317) and two downregulated (miR-100-5p, miR-133a) (p<0.05) miRNA genes. Four of these miRNA were already reported in HNSCC: miR-21-5p and miR-21-3p [11, 27–29], miR-100 [27, 30], miR-133a [28]. Three miRNAs namely miR-1246, miR-1290 and miR-4317, which were recurrently overexpressed both in the LSCC cell lines and in the primary tumors and were not reported in HNSCC, were regarded as the best candidates for potential oncomiRs and further analyzed.

**Table 2:** miRNAs deregulated in LSCC cell lines and primary samples.

*Target ID*	*Agilent Probe ID*	*Fold change*, *compared to no tumor controls*, *in*		*Accession number*	*Chromosomal location (GRCh37/hg19)*
		*LSCC cell lines*	*Primary tumor cases*		
hsa-miR-1290	**A_25_P00015107**	14.268	3.275	MIMAT0005880	chr1: 19223565–19223642 [–]
hsa-miR-1246	**A_25_P00015143**	11.717	4.523	MIMAT0005898	chr2: 177465708–177465780 [–]
hsa-miR-21-5p	**A_25_P00010975**	5.134	4.268	MIMAT0000076	chr17: 57918627–57918698 [+]
hsa-miR-21-5p	**A_25_P00010976**	3.839	4.216	MIMAT0000076	chr17: 57918627–57918698 [+]
hsa-miR-21-3p	**A_25_P00013174**	4.335	4.061	MIMAT0004494	chr17: 57918627–57918698 [+]
hsa-miR-4317	**A_25_P00015487**	2.212	2.021	MIMAT0016872	chr18: 6374360–6374424 [–]
hsa-miR-133a	**A_25_P00012166**	-2.422	-2.667	MIMAT0000427	chr18: 19405659–19405746 [–]
hsa-miR-100-5p	**A_25_P00010474**	-3.373	-3.809	MIMAT0000098	chr11: 122022937–122023016 [–]

MiRNAs significantly altered in both groups (LSCC cell lines and primary tumor cases) compared to no tumor controls (Agilent Human miRNA Microarray Expression 60K platform).

### LNA real-time qPCR validates overexpression of miR-1246 and miR-1290 but not miR-4317 in LSCC

**Fig 1:**
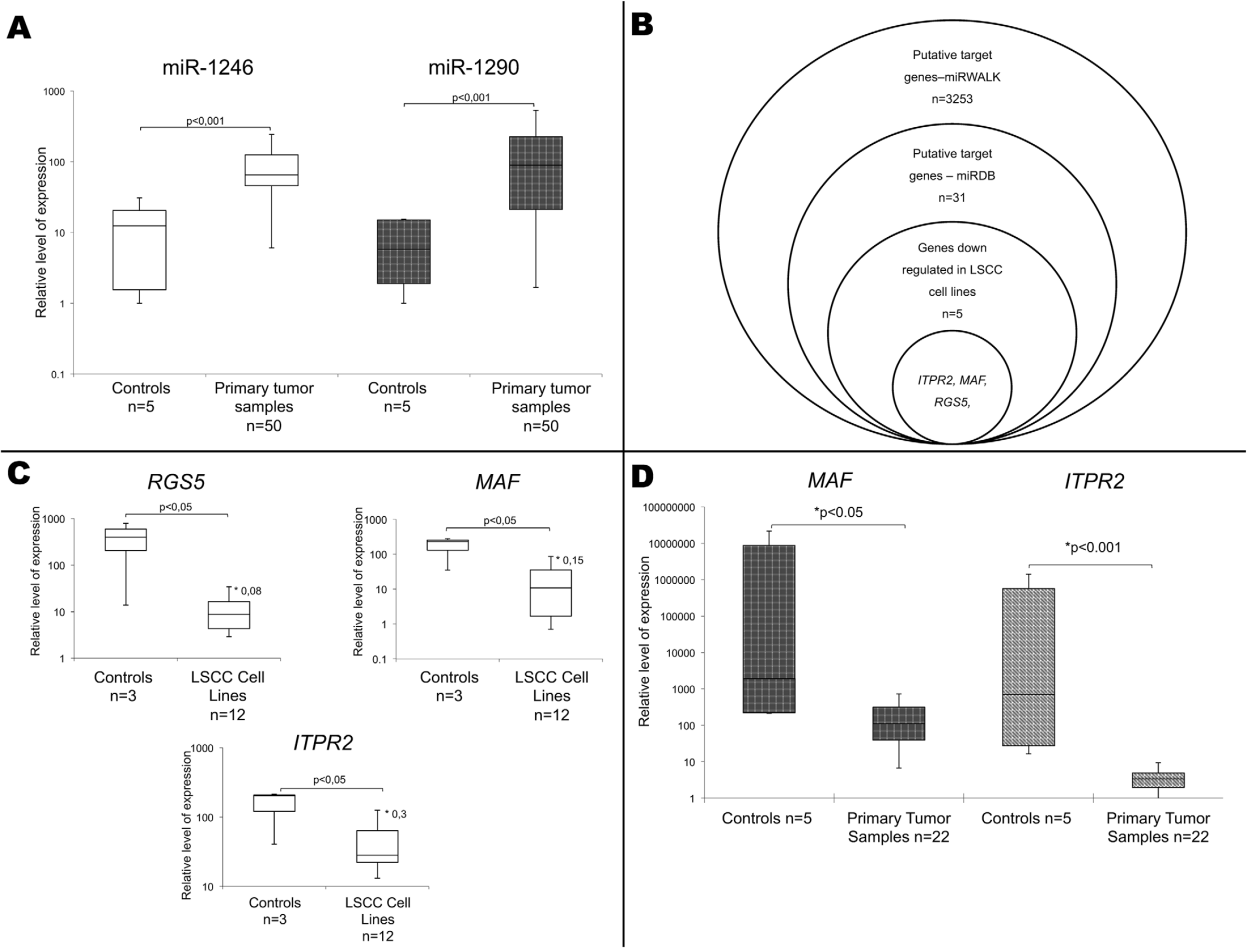
Expression level of selected miRNA and selection scheme of miR-1290 target genes. A) Overexpression of miR-1290 and miR-1246 in primary LSCC cases (LNA real-time qPCR). B) Schematic presentation of the selection process of miR-1290 candidate genes. C) Expression level of *MAF*, *ITPR2* and *RGS5* in LSCC cell lines and no tumor controls (Affymetrix U133 plus 2.0) based on [20]. * fold change in LSCC cell lines compared to no tumor controls. D) Expression level of *MAF* and *ITPR2* in 22 primary tumor samples compared to 5 no tumor controls.

In order to validate the microarray miRNA profiles the expression of these three candidates was analyzed using LNA quantitative real-time PCR. We confirmed overexpression of miR-1246 (mean relative expression 311.4 and SD 52.4) and miR-1290 (mean relative expression 48.4; SD 10.84) in an independent cohort of 50 primary LSCC specimens as compared to 5 epithelial no tumor controls (mean relative expression 16.56 and 7.83; SD 2.95 and 1.27 for miR-1246 and miR-1290 respectively) with fold change of 18.8 for miR-1246 and 25.9 for miR-1290 (Fig 1A). The differences were highly significant (Mann Whitney U-test, p<0.001). In case of miR-4317 no differences were detected. While conducting this study, miR-1246 was reported to be a pseudogene [31], therefore only miR-1290 was selected for further analysis.

### miR-1290 regulates genes potentially implicated in LSCC

To elucidate the biological significance of miR-1290 overexpression in LSCC we performed data-mining using the miRDB database and miRWALK software as described in the materials and methods section. We have identified 31 genes potentially regulated by miR-1290, out of which 5 genes were statistically significant downregulated [fold change ≤ 0.6(6)] in LSCC cell lines compared to 3 epithelial no tumor controls (Fig 1B). Out of these, we have chosen three most changed in LSCCs namely *RGS5* (fold change 0.08), *MAF* (fold change 0.15), and *ITPR2* (fold change 0.3) which are attractive candidates for novel tumor suppressors according to their function and involvement in other cancers (Fig 1C).

To verify if the observed downregulation of *MAF*, *ITPR2* and *RGS5* was characteristic also for primary LSCC specimens, we analyzed group of 22 tumors and five no tumor controls by quantitative real time PCR. We observed statistically significant downregulation of *ITPR2* (p<0.001; mean relative expression 3.78 in LSCC samples and 1.7x10^7^ for no tumor controls) and *MAF* (p<0.05; mean relative expression 310.14 in LSCC samples and 1.6x10^7^ for no tumor controls) but not *RGS5* (p>0.05; mean relative expression 1266.25 in LSCC samples and 1888.96 for no tumor controls) (Fig 1D).

To verify that miR-1290 overexpression is responsible indeed for the observed downregulation of *ITPR2* and *MAF* we transfected the UT-SCC-34 and UT-SCC-107 cell lines with the miR-1290 inhibitor or the inhibitor negative control and analyzed the changes of expression of these target genes.

**Fig 2:**
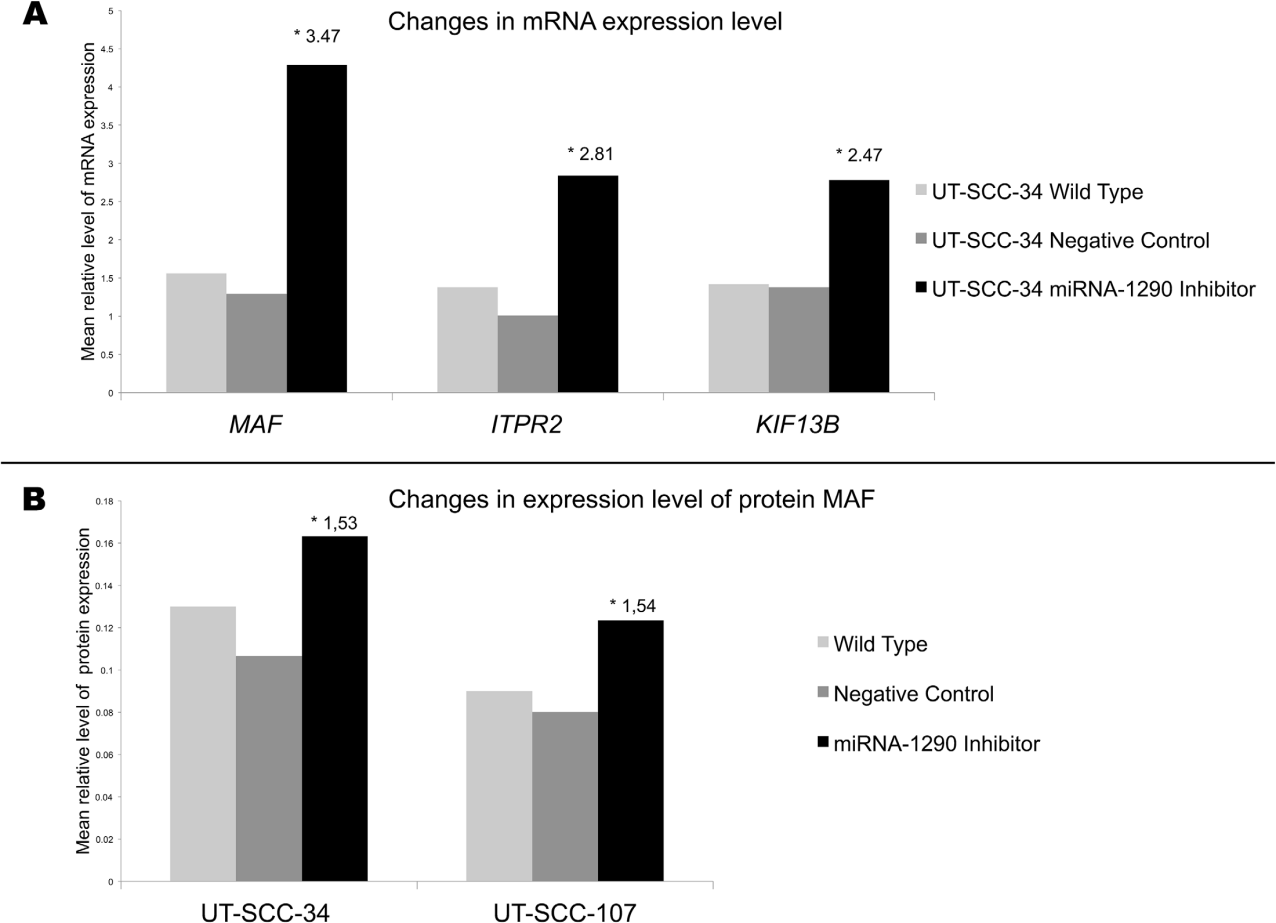
Expression level of miR-1290 target genes. A) Changes in expression level of *MAF*, *ITPR2* and *KIF13B* genes in UT-SCC-34 treated by miR-1290 inhibitor (black graph) compared to both UT-SCC-34 treated by negative control (dark gray graph) as well as UT-SCC-34 wild type (gray graph). * fold change in UT-SCC-34 treated by miR-1290 inhibitor compared to UT-SCC-34 treated by negative control. B) Changes in expression level of MAF protein in UT-SCC-34 and UT-SCC-107cell lines treated by miR-1290 inhibitor (black graph) compared to both cells treated by negative control (dark gray graph) as well as wild type cells (gray graph). * fold change in cells treated by miR-1290 inhibitor compared to cells treated by negative control.

In line with our hypothesis, we observed an increased mRNA expression of the selected genes (*MAF*—fold change 3.47 and *ITPR2*—fold change 2.81; Fig 2A) in the probes treated by the inhibitor compared to the inhibitor negative control in the UT-SCC-34 cell line (UT-SCC-107 not analyzed). In aim to verify the transfection system additionally we analyzed expression level of the known miR-1290 target gene—*KIF13B* and similarly observed increased expression of this gene after inhibition (fold change 2.47).

Moreover, we confirmed these findings on protein level for MAF. In the probes treated by the inhibitor compared to the inhibitor negative control an increased expression of 53% in UT-SCC-34 and 54% in UT-SCC-107 was observed (Fig 2B). Whereas, the Western blot analysis for ITPR2 did not detect any protein product for this gene in the analyzed cell lines while for HepaRG (positive control of the antibody) the proper band was present. Thus the results for ITPR2 were not conclusive.

## Discussion

In the present study we performed genome-wide miRNA expression profiling and identified 5 miRNAs (miR-21-3p, miR-21-5p, miR-1246, miR-1290, miR-4317), which showed significant overexpression and two downregulated (miR-100-5p, miR-133a) in LSCC cell lines and primary tumors as compared to no tumor controls. MiR-21 has already been reported to regulate cell migration and tumorigenicity and to be overexpressed in LSCC [29, 32, 33]. MiR-100-5p and miR-133a were also reported in LSCC before [27,28, 30]. Thus, our data provide further evidence for its role in the pathogenesis of this cancer. For the three remaining miRNAs, we validated the overexpression of miR-1246 and miR-1290 but not miR-4317 with an independent method in primary laryngeal cancer specimens. As miR-1246 has been reported a pseudogene [31], which certainly requires further confirmation, we have chosen only miR-1290 for further analyses.

MiR-1290 was already reported in several cancers, including cervical, colon, hepatocellular, prostate and renal malignancies [34–38]. Overexpression of miR-1290 was demonstrated as one of the causes of multinucleated cell formation which leads to cellular reprogramming in colon cancer [34]. The delay in cytokinesis was induced by downregulation of miR-1290 target gene *KIF13B* which is part of the Wnt pathway. There are no reports about the role of *KIF13B* in HNSCC but alterations of other components of the Wnt pathways were described [39–41]. Additionally, overexpressed miR-1290 is involved in radio-resistance of cervical cancer, both in adenocarcinoma as well as in squamous cell carcinoma [35], which is common among LSCC patients [42]. Interestingly, miR-1290 was also found downregulated in HepG2 cells treated with paclitaxel [36], well known chemotherapeutic, also tested in LSCC [43]. Paclitaxel is known to induce cell death [44], and thus decreased miR-1290 expression after treatment might implicate involvement of this miRNA in apoptosis inhibition.

In light of the discussed reports miR-1290 is suggested to have oncogenic potential in various types of cancer. Noteworthy, during the cause of this study miR-1290 was reported overexpressed in supraglottic LSCC by Sun et.al. [18] providing another argument for the importance of miR-1290 in the development of LSCC.

In our study in addition to showing deregulation of miR-1290 in LSCC we delineated a group of its target genes among which two, namely, *MAF* and *ITPR2* were significantly downregulated in primary LSCC specimens. According to the data presented these are interesting candidates for novel tumor suppressor genes in LSCC.

*MAF* (*c*-*MAF*) transcription regulator, belongs to the evolutionally conserved family of large MAF proteins, which are involved in terminal differentiation of tissues [45] and thus may be related to the observed de-differentiation of epithelial cells in SCC. Depending on occurring abnormalities in cancer MAF could act as suppressor or oncogene. Moreover, in mice Maf was shown to regulate apoptosis by activation of Caspase 6 [46] and the tp53 [47]–both known phenomena deregulated in HNSCC [42, 48]. *ITPR2* in turn, known as an important factor in Ca^2+^ mobilization from endoplasmic reticulum (ER) [48], may similarly impact apoptosis regulation [49]. Interestingly, ER Ca^2+^ release leads moreover to senescence of cells [50], which in HNSCC was shown to sensitize the tumor to radiotherapy [51]. The involvement of *MAF* and *ITPR2* in the regulation of apoptosis and radioresistance reflects therefore well the role of miR-1290 reported in other cancers. Noteworthy, none of the genes was reported in the context of LSCC so far.

In our study, we modulated the expression of miR-1290 in UT-SCC-34 and UT-SCC-107 cell lines in order to demonstrate functionally its involvement in regulation of *MAF* and *ITPR2*. Along with our hypothesis, we observed these genes to be regulated by miR-1290 on mRNA level. We also demonstrated increased level of MAF protein in cells after miR-1290 inhibition. The absence of ITPR2 protein after miR-1290 inhibition indicates a more complicated mechanism of its expression regulation. Although we have noticed higher expression of this gene on mRNA level after inhibition of miR-1290 there was no increase of the respective protein. One possible explanation is that the ITPR2 gene could be blocked by other miRNAs in the tumor cells. Both genes are interesting candidates for novel tumor suppressors deregulated LSCC pathogenesis but the full understanding of their role requires further studies.

In conclusion, our results suggest the involvement of miR-1290 in LSCC development. Moreover, we show that the pathogenic potential of miR-1290 may be manifested through the downregulation of its target genes *MAF* and *ITPR2*.

## Supporting Information

S1 Table Primer sequences used in quantitative real-time PCR. (DOCX)

S2 Table miRNA expression microarray results. MiRNAs significantly altered (at least two fold change) in group of LSCC cell lines and primary cases respectively, compared to controls (Agilent Human miRNA Microarray Expression 60K platform).
(DOCX)
