# Supplementary material for: Global miRNA Expression Profiling Identifies miR-1290 as Novel Potential oncomiR in Laryngeal Carcinoma
Source: PLoS One. 2015 Dec 22;10(12):e0144924. doi: 10.1371/journal.pone.0144924 (PMC4692263; doi:10.1371/journal.pone.0144924)
Supplement: S1 Table — (DOCX) [file pone.0144924.s001.docx]

**S1 Table. Primer sequences used in quantitative real-time PCR**.

| Gene | Forward primers | Reverse primers | Product lenght | Tm | Efficiency |
| --- | --- | --- | --- | --- | --- |
| *β-ACTIN* | CACCACACCTTCTACAATG | TAGCACAGCCTGGATAG | 162 | 60 | 101% |
| *EMP2* | TTGTCCTAACCTCCATCAT | CTGGTCACGGGATAGAAT | 121 | 55 | 87% |
| *ITPR2* | AATACAGTAATGTTATACAACTAC | TTCCAGAACGGATGAATA | 157 | 54 | 90% |
| *MAF* | AATACGAGAAGTTGGTGA | TTTGTGAACACACTGGTA | 169 | 55 | 108% |
| *RGS5* | ACCTTGAGTTCTGGATTG | TTGTGATGTCCTTAGTGAA | 150 | 55 | 98% |
| *RORA* | CACCGAGAAGATGGAATACTA | TGAAGTCGCACAATGTCT | 125 | 54 | 99% |
| *KIF13B* | GGAGCAACATTAACAAGT | CTGAGTCACGATATGGAA | 108 | 51 | 100% |
