# Supplementary material for: Global miRNA Expression Profiling Identifies miR-1290 as Novel Potential oncomiR in Laryngeal Carcinoma
Source: PLoS One. 2015 Dec 22;10(12):e0144924. doi: 10.1371/journal.pone.0144924 (PMC4692263; doi:10.1371/journal.pone.0144924)
Supplement: S2 Table — MiRNAs significantly altered (at least two fold change) in group of LSCC cell lines and primary cases respectively, compared to controls (Agilent Human miRNA Microarray Expression 60K platform). (DOCX) [file pone.0144924.s002.docx]

**S2 Table miRNA expression microarray results.** miRNAs significantly altered (at least two fold change) in group of LSCC cell lines and primary cases respectively, compared to controls (Agilent Human miRNA Microarray Expression 60K platform).

| ***Target ID*** | ***Agilent Probe ID*** | ***P-value*** | ***Log (fold change)*** | ***Fold change*** | ***Accession number*** |
| --- | --- | --- | --- | --- | --- |
| **Laryngeal squamous cell carcinoma lines vs. controls** | | | | | |
| hsa-miR-1290 | **A_25_P00015107** | 0,003657082 | 3,834697675 | 14,26786609 | MIMAT0005880 |
| hsa-miR-196b-5p | **A_25_P00012412** | 0,034743609 | 3,664040483 | 12,67611267 | MIMAT0001080 |
| hsa-miR-1246 | **A_25_P00015143** | 0,000943472 | 3,550533334 | 11,7170163 | MIMAT0005898 |
| hsa-miR-7-5p | **A_25_P00012078** | 0,04649668 | 3,442828956 | 10,87413664 | MIMAT0000252 |
| hsa-miR-196a-5p | **A_25_P00012053** | 0,018711607 | 3,211784811 | 9,264960405 | MIMAT0000226 |
| hsa-miR-141 | **A_25_P00010548** | 2,07914E-06 | 2,761249054 | 6,779829787 | MIMAT0000432 |
| hsa-miR-106b-5p | **A_25_P00010434** | 0,003154145 | 2,562360218 | 5,906732256 | MIMAT0000680 |
| hsa-miR-96-5p | **A_25_P00012034** | 5,3055E-09 | 2,528395585 | 5,769297203 | MIMAT0000095 |
| hsa-miR-106b-5p | **A_25_P00010433** | 0,035083798 | 2,451398695 | 5,469461106 | MIMAT0000680 |
| hsa-miR-196b-5p | **A_25_P00012411** | 0,008704221 | 2,434320811 | 5,405098132 | MIMAT0001080 |
| hsa-miR-21-5p | **A_25_P00010975** | 0,02247934 | 2,360015859 | 5,133760023 | MIMAT0000076 |
| hsa-miR-96-5p | **A_25_P00012035** | 1,49421E-08 | 2,354783835 | 5,115175838 | MIMAT0000095 |
| hsa-miR-93-5p | **A_25_P00010611** | 0,020970259 | 2,279883932 | 4,856388813 | MIMAT0000093 |
| hsa-miR-21-3p | **A_25_P00013174** | 0,019516338 | 2,116023964 | 4,334975872 | MIMAT0004494 |
| hsa-miR-141-3p | **A_25_P00010547** | 0,005315714 | 2,082904117 | 4,236591762 | MIMAT0000432 |
| hsa-miR-192-5p | **A_25_P00010868** | 0,04658512 | 1,997859482 | 3,994069624 | MIMAT0000222 |
| hsa-miR-25-3p | **A_25_P00010990** | 0,000134791 | 1,957452022 | 3,883754538 | MIMAT0000081 |
| hsa-miR-454-3p | **A_25_P00012871** | 0,000282115 | 1,951115928 | 3,866735087 | MIMAT0003885 |
| hsa-miR-361-5p | **A_25_P00013981** | 0,009818763 | 1,940699608 | 3,838917638 | MIMAT0000703 |
| hsa-miR-21-5p | **A_25_P00010976** | 0,011536814 | 1,940566138 | 3,8385625 | MIMAT0000076 |
| hsa-miR-98 | **A_25_P00010047** | 2,22789E-06 | 1,931054004 | 3,813336922 | MIMAT0000096 |
| hsa-miR-151a-3p | **A_25_P00014020** | 0,026779535 | 1,879376366 | 3,679159868 | MIMAT0000757 |
| hsa-miR-30a-3p | **A_25_P00010827** | 0,031938262 | 1,812931007 | 3,513553842 | MIMAT0000088 |
| hsa-miR-324-5p | **A_25_P00010153** | 0,000971187 | 1,807096009 | 3,49937193 | MIMAT0000761 |
| hsa-miR-151a-5p | **A_25_P00012375** | 0,029664292 | 1,718501633 | 3,290944356 | MIMAT0004697 |
| hsa-miR-625-5p | **A_25_P00012810** | 0,021414736 | 1,595303583 | 3,021580929 | MIMAT0003294 |
| hsa-miR-324-5p | **A_25_P00010154** | 7,71259E-05 | 1,594253528 | 3,019382494 | MIMAT0000761 |
| hsa-miR-15b-5p | **A_25_P00011102** | 0,012470396 | 1,575521022 | 2,9804311 | MIMAT0000417 |
| hsa-miR-25-3p | **A_25_P00010989** | 0,028109968 | 1,529855447 | 2,887569052 | MIMAT0000081 |
| hsa-miR-374b-5p | **A_25_P00014915** | 0,038609921 | 1,521133125 | 2,870163902 | MIMAT0004955 |
| hsa-miR-30a-3p | **A_25_P00014976** | 0,023977462 | 1,394577799 | 2,629116003 | MIMAT0000088 |
| hsa-miR-15b-5p | **A_25_P00011101** | 0,000539096 | 1,367628937 | 2,580461197 | MIMAT0000417 |
| hsa-miR-16-2-3p | **A_25_P00013271** | 0,033544408 | 1,331620259 | 2,516851784 | MIMAT0004518 |
| hsa-miR-151a-5p | **A_25_P00012376** | 1,99069E-05 | 1,277203557 | 2,423687267 | MIMAT0004697 |
| hsa-miR-107 | **A_25_P00011069** | 0,045693249 | 1,255690186 | 2,387813543 | MIMAT0000104 |
| hsa-miR-135b-5p | **A_25_P00012382** | 0,034628385 | 1,224782884 | 2,337202738 | MIMAT0000758 |
| hsa-miR-4317 | **A_25_P00015487** | 0,01291571 | 1,145543812 | 2,212295057 | MIMAT0016872 |
| hsa-miR-98 | **A_25_P00010048** | 0,037621171 | 1,125591102 | 2,181909256 | MIMAT0000096 |
| hsa-miR-135b-5p | **A_25_P00012383** | 0,043547095 | 1,082244809 | 2,117328045 | MIMAT0000758 |
| hsa-miR-30d-5p | **A_25_P00010683** | 0,043693489 | 1,058794771 | 2,083190495 | MIMAT0000245 |
| hsa-let-7f-5p | **A_25_P00010089** | 0,01691715 | 1,048364847 | 2,068184435 | MIMAT0000067 |
| hsa-miR-148b-3p | **A_25_P00010133** | 0,002523552 | 1,041187234 | 2,057920477 | MIMAT0000759 |
| hsa-miR-671-5p | **A_25_P00012860** | 0,003104446 | -1,062879251 | -2,089096668 | MIMAT0003880 |
| hsa-miR-133a | **A_25_P00012166** | 0,032325628 | -1,276046698 | -2,421744554 | MIMAT0000427 |
| hsa-miR-4299 | **A_25_P00015749** | 0,000548862 | -1,414577128 | -2,665815855 | MIMAT0016851 |
| hsa-miR-4299 | **A_25_P00015750** | 0,001460866 | -1,422755809 | -2,680971362 | MIMAT0016851 |
| hsa-let-7b-5p | **A_25_P00010071** | 0,001670221 | -1,580876609 | -2,991515647 | MIMAT0000063 |
| hsa-miR-100-5p | **A_25_P00010474** | 0,020734812 | -1,754109112 | -3,373179547 | MIMAT0000098 |
| hsa-miR-125b-5p | **A_25_P00010980** | 0,004290672 | -1,905329379 | -3,74594412 | MIMAT0000423 |
| hsa-let-7c | **A_25_P00010073** | 0,004218328 | -1,924836938 | -3,796939307 | MIMAT0000064 |
| hsa-miR-940 | **A_25_P00013090** | 0,006323136 | -2,06424259 | -4,18214357 | MIMAT0004983 |
| hsa-let-7c | **A_25_P00010072** | 0,000459966 | -2,228205558 | -4,685508278 | MIMAT0000064 |
| hsa-miR-125b-5p | **A_25_P00010979** | 0,023527236 | -2,483431015 | -5,592258363 | MIMAT0000423 |
| **LSCC primary cases vs. Controls** | | | | | |
| hsa-miR-1246 | **A_25_P00015143** | 0,011274361 | 2,177334909 | 4,52317216 | MIMAT0005898 |
| hsa-miR-21-5p | **A_25_P00010976** | 0,003986183 | 2,093618 | 4,268171065 | MIMAT0000076 |
| hsa-miR-155-5p | **A_25_P00012270** | 0,004940221 | 2,08704939 | 4,248782198 | MIMAT0000646 |
| hsa-miR-21-5p | **A_25_P00010975** | 0,037901179 | 2,075883493 | 4,216025208 | MIMAT0000076 |
| hsa-miR-21-3p | **A_25_P00013174** | 0,019864751 | 2,0219013 | 4,061186553 | MIMAT0004494 |
| hsa-miR-155-5p | **A_25_P00012271** | 0,012208504 | 1,767977621 | 3,405762 | MIMAT0000646 |
| hsa-miR-1290 | **A_25_P00015107** | 0,037292854 | 1,711381292 | 3,274742098 | MIMAT0005880 |
| hsa-miR-361-3p | **A_25_P00012305** | 0,008370517 | 1,365079223 | 2,575904707 | MIMAT0004682 |
| hsa-miR-4317 | **A_25_P00015487** | 0,030190512 | 1,015209857 | 2,021196878 | MIMAT0016872 |
| hsa-miR-133a | **A_25_P00012166** | 0,032540343 | -1,415139856 | -2,666855868 | MIMAT0000427 |
| hsa-miR-100-5p | **A_25_P00010474** | 0,024895635 | -1,929581081 | -3,809445674 | MIMAT0000098 |
| hsa-miR-30a-5p | **A_25_P00013852** | 0,036776063 | -1,945230235 | -3,850992292 | MIMAT0000087 |
| hsa-miR-30a-5p | **A_25_P00013853** | 0,016057983 | -2,157339102 | -4,460913278 | MIMAT0000087 |
| hsa-miR-100-5p | **A_25_P00010475** | 0,026480423 | -2,511588891 | -5,702477667 | MIMAT0000098 |
